# Role of Exosomes in the Regulation of T-Cell Mediated Immune Responses and in Autoimmune Disease

**DOI:** 10.3390/cells8020154

**Published:** 2019-02-12

**Authors:** Alberto Anel, Ana Gallego-Lleyda, Diego de Miguel, Javier Naval, Luis Martínez-Lostao

**Affiliations:** 1 Immunity, Cancer & Stem Cells Group, Department of Biochemistry and Molecular and Cell Biology, Faculty of Sciences, Campus San Francisco Sq., University of Zaragoza and Aragón Health Research Institute (IIS Aragón), E-50009 Zaragoza, Spain; jnaval@unizar.es; 2 Department of Biochemistry and Molecular and Cell Biology, Faculty of Sciences, Campus San Francisco Sq., University of Zaragoza and Aragón Health Research Institute (IIS Aragón), E-50009 Zaragoza, Spain; annaiss89@hotmail.com; 3 Centre for Cell Death, Cancer and Inflammation (CCCI), UCL Cancer Institute, University College London, Gower St, Bloomsbury, London WC1E 6BT, UK; diego_demiguel@hotmail.com; 4 Immunology Department, Lozano Blesa Clinical Hospital, and Aragón Health Research Institute (IIS Aragón), E-50009 Zaragoza, Spain; lmartinezlos@salud.aragon.es

**Keywords:** exosomes, extracellular vesicles, immune regulation, autoimmunity

## Abstract

T-cell mediated immune responses should be regulated to avoid the development of autoimmune or chronic inflammatory diseases. Several mechanisms have been described to regulate this process, namely death of overactivated T cells by cytokine deprivation, suppression by T regulatory cells (Treg), induction of expression of immune checkpoint molecules such as CTLA-4 and PD-1, or activation-induced cell death (AICD). In addition, activated T cells release membrane microvesicles called exosomes during these regulatory processes. In this review, we revise the role of exosome secretion in the different pathways of immune regulation described to date and its importance in the prevention or development of autoimmune disease. The expression of membrane-bound death ligands on the surface of exosomes during AICD or the more recently described transfer of miRNA or even DNA inside T-cell exosomes is a molecular mechanism that will be analyzed.

## 1. Mechanisms of Immune T Cell Tolerance

The maintenance of immune homeostasis is dependent on immune tolerance towards self-tissues and is a complex process, necessary to avoid autoimmunity. In the case of T cells, two types of tolerance are needed, central and peripheral tolerance. Central tolerance takes place during thymic maturation, achieving the deletion of autoreactive immature thymocytes, a process also known as negative selection [1]. Peripheral tolerance comprises several mechanisms acting on mature T cells in peripheral tissues or circulation [2]. Among the known T-cell peripheral tolerance mechanisms are the following:

(i) If the antigen is presented by cells that are not professional antigen-presenting cells (APC), or by immature APC, they do not provide co-stimulation signals and induce T cell anergy [3,4,5].
(ii) The immunosuppressive activity of regulatory T cells (Treg) [6].
(iii) The regulated termination of T cell immune responses [7], which, in turn, is dependent on several complex mechanisms. In fact, other possible mechanisms could still be discovered.

On one hand, T cell activation results in the induction of the expression of negative regulators of its own activation, the so-called immune checkpoints. The first checkpoint molecule to be described was CTLA-4 [8]. CLTA-4 competes with CD80/CD86 for the T cell co-stimulator CD28 [9], and, in addition, transmit inhibitory signals inside T cells [10]. Immune regulation by CTLA-4 is important since CTLA-4 knockout mice develop fatal lymphoproliferative disorders [11] and mutations in the CTLA-4 gene have been associated in humans with an increased risk of autoimmune disease [12,13]. Another important checkpoint molecule is PD-1 [14], which is also expressed on the surface of T cells upon activation, and that, by binding to its ligands PD-L1 and PD-L2, activate tyrosine phosphatase activities promoting the turning off of tyrosine kinase-mediated activating signals [15]. This mechanism is important to down-modulate inflammation in peripheral tissues in a physiological manner [16]. The use of blocking anti-CTLA-4 and anti-PD-1 antibodies in the immunotherapy of cancer has given excellent results, and this has been recognized with the Nobel Prize 2018 granted to the pioneers in the field, Jim P. Allison and Tasuku Honjo [17]. Other immune checkpoint molecules that regulate immune function are LAG-2, TIM-3 or TIGIT [18].

On the other hand, the deprivation of immuno-stimulatory cytokines such as IL-7, IL-2 and IL-15 due to T cell migration to peripheral tissues from spleen or lymph nodes is the main cause of down-modulation of T cell responses, especially those mediated by CD8^+^ T cells, unable to produce their own cytokines [19]. Bim, a BH3-only, pro-apoptotic member of the Bcl-2 family, is the main regulator of this process, and defects in its expression are associated with autoimmunity [20,21].

Finally, the termination of immune responses is also mediated by activation-induced cell death (AICD) of T cells. The main regulator of AICD is the Fas/Fas ligand (FasL) system [22,23], and mutations in Fas or FasL are the cause of the autoimmune lympho-proliferative syndromes (ALPS) [24]. Apo2L/TRAIL (Apo2 Ligand/TNF-related apoptosis-inducing ligand) is another member of the FasL death ligand family and it has also been implicated in human T cell AICD [25,26]. It rather functions as a fine-tuning modulator of IL2-dependent CD8^+^ T cell proliferation [27] or in the elimination of CD8^+^ T cells activated in the absence of CD4^+^ T cell help [28]. No autoimmune disease is known to be associated with TRAIL mutations, although TRAIL-knockout mice are more sensitive to the induction of experimental autoimmune diseases [29].

## 2. Exosomes in Immune Regulation

### 2.1. Exosomes in Immune Cells

Exosomes are secreted extracellular membrane vesicles, with a particular lipid and protein composition, and size between 30 and 120 nm [30]. These exosomes are stored in cytoplasmic multivesicular bodies as intraluminal vesicles before secretion. A wide range of cell types are able to secrete exosomes such as melanocytes [31], platelets [32], trophoblasts [33], intestinal, prostate and intraocular epithelial cells [34,35,36], and, of course, also immune cells such as dendritic cells [37,38], B lymphocytes [39], T lymphocytes [40,41], neutrophils [42] and mast cells [43]. In addition, exosomes are present in blood plasma [44], colon mucosa [45], in lactating mammary glands and milk [46,47], human urine [48] and human bronco alveolar fluid [49]. On the other hand, exosome secretion has been also described in different types of tumor cells, and it has been proposed to play an important role in tumorigenesis and metastasis [50,51].

**Figure 1:**
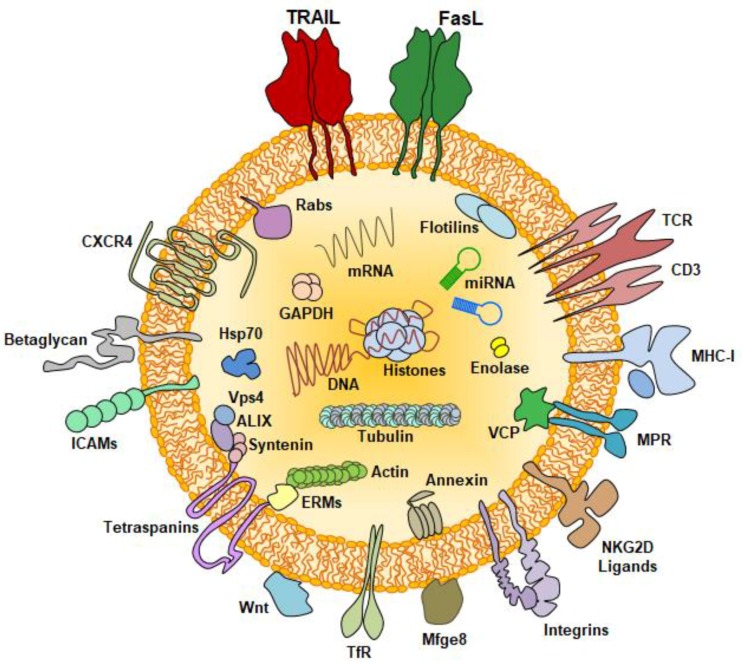
Schematic representation of a T cell derived exosome, showing the location of several of the most important functional proteins expressed, together with miRNA and DNA.

Regarding exosomes produced by activated T cells, proteomic and immunoblot studies [52,53] have shown the expression of proteins present in most exosomes, such as the membrane tetraspanins CD63 and CD81, annexins and major luminal proteins such as actin and tubulin isoforms, specific heat-shock proteins, enolase and GAPDH, but also of proteins related with immune function such as HLA-I, β2-microglobulin, components of the TCR/CD3 complex and specific integrins, among others (see Figure 1). Of note, the membrane-associated ATPase VCP has been detected in exosomes from leukemic T cells, but not in exosomes of T cells from healthy donors [52]. Other functional components of exosomes are regulatory miRNA [54], and in T cell exosomes it has been demonstrated that the enrichment in specific miRNA in T cell exosomes is dependent on the activity of another major exosomal protein, the heterogeneous nuclear riboprotein A2/B1 [55]. More recently, the presence of DNA inside T cell exosomes has been described [56].

For a complete repository of proteins and miRNA expressed in exosomes see also the Exocarta and Vesiclepedia websites http://exocarta.org/index.html; http://www.microvesicles.org.

Exosomes produced by immune cells play a role in the activation of immune responses in many instances. In this line, exosomes secreted by dendritic cells and B cells, which express MHC-I and MHC-II on their surface, act as antigen-presenting platforms and participate in T cell priming and activation [39,57]. In addition, the unidirectional transfer of miRNA from T cells to antigen-presenting cells has been demonstrated, contributing and/or regulating the final outcome of T cell activation [58,59].

### 2.2. Role of Exosomes in AICD and in Pregnancy and Lactation

Although immune exosomes play a role in T cell activation, as previously mentioned, their role in immune regulation processes has been more extensively studied, mainly in the context of death ligand-mediated T cell AICD. Although it was initially reported that the soluble form of FasL, generated through the metalloproteinase-mediated cleavage of the membrane protein, retained its cytotoxic potential [60,61], later studies demonstrated that FasL release in its soluble form due to the action of metalloproteases is a mechanism of functional down-regulation [62,63,64]. In addition, Fas and TRAIL receptors are physiologically expressed in the cell surface as pre-assembled oligomeric complexes, forming homo-trimers [65,66,67]. These complexes are formed through interactions of specific extracellular cysteine-rich domains called PLAD (pre-ligand assembly domain) [67]. Congruent with this, a potent pro-apoptotic activity of death ligands is dependent on the oligomerization of death receptor trimers in supramolecular structures [65,68]. Physiologically, this can only be achieved if death ligands are displayed on membrane structures: on the plasma membrane of effector cells [69], or on the surface of extracellular vesicles [70].

Our group described that both FasL and Apo2L/TRAIL are stored inside human T cell blasts in multivesicular bodies [71], being rapidly released to the supernatant in their bioactive form, associated with exosomes, upon T cell re-activation [70,71]. This observation was confirmed later on by other groups [72,73,74]. Death ligands secreted in this membrane-bound form fully conserve their death receptor cross-linking efficiency, correlating with their pro-apoptotic potential, thus efficiently participating in the down-modulation of T cell-mediated immune responses. In addition, a similar immunoregulatory role has been also described for exosomes produced by activated human NK cells [75].

Data obtained in mice knockout for the Wiskott-Aldrich syndrome (WAS) protein or in cytotoxic T lymphocyte (CTL) clones derived from Chediak-Higashi syndrome (CHS) patients gave some cues to demonstrate the physiological relevance of this mechanism. WAS and CHS are both primary immune-deficiencies, but they usually progress to autoimmunity. In the case of the WAS knockout mice, it was demonstrated that autoimmunity manifestations were due to the absence of functional FasL secretion associated with exosomes from T cells [76]. A similar situation was observed in CTL clones derived from CHS patients, that, although able to express FasL on their plasma membrane, were unable to secrete FasL associated with exosomes [77].

In other contexts, it has been described that T cell exosomes expressing FasL also down-modulate dendritic cell activation, leading to the termination of immune responses [78]. In addition, circulating extracellular vesicles (EVs) have an immunosuppressive activity [79,80] and this activity is also dependent on the expression of FasL [81]. This mechanism could be important in preventing self and foreign antigens from causing chronic inflammation and autoimmunity.

Another physiological setting in which exosome-mediated immune regulation is relevant is during the development of maternal-fetal tolerance. It has been described that FasL is secreted on the surface of exosomes by trophoblasts, accomplishing an important function in the attenuation of the immune response against the fetus and preventing spontaneous abortion [33,82]. In fact, it has been demonstrated that EVs derived from the serum of pregnant mice prevent further central nervous system injury in established experimental autoimmune encephalomyelitis [83].

Finally, EVs isolated from breast milk promote Treg development and proliferation, favoring tolerance processes [46,84].

### 2.3. Exosomes in Other Immune Regulatory Mechanisms

The implication of exosome release in the other immune regulatory mechanisms described in Section 1 is less studied, but some reports reveal its importance. Noteworthy, it has been demonstrated that regulatory T cells (Treg) actively release immunosuppressive exosomes that inhibit IFN-γ secretion and the proliferation of Th1 effector cells [85]. In addition, Treg-derived exosomes induce the differentiation of other T cells to the Treg phenotype [86]. In this line, tumor exosomes, and probably other immune cell-derived exosomes, induce the differentiation of monocytes to monocyte-derived suppressor cells (MDSC), which suppress T cell proliferation and function [87]. Similarly, membrane PD-L1 [88,89] and the immunosuppressive cytokine TGF-β [90] have been found recently in tumor-derived exosomes, but their presence in exosomes mediating physiological tolerance processes has not been described yet.

Finally, EVs from endothelial cells also modulate T cell responses and prevent chronic inflammation in tissues, in this case through the transfer of anti-inflammatory miRNA [91], and mesenchymal stem cells secrete immunosuppressive exosomes, which in fact are being used in clinical trials to prevent autoimmunity [92,93,94].

## 3. Exosomes in Autoimmune and Chronic Inflammatory Diseases

### 3.1. Exosomes in Rheumatoid Arthritis and Joint Diseases

In general, exosomes produced by inflammatory infiltrated cells are pathogenic in rheumatoid arthritis (RA) and other joint diseases [95,96]. Exosomes produced by synoviocytes in an inflammatory environment stimulate articular cells to secrete more inflammatory mediators and degradative enzymes, contributing to cartilage damage [97,98,99]. In addition, exosomes located in the synovium of RA patients, probably produced by proliferating synoviocytes, contain citrullinated autoantigens and promote inflammation [100,101]. On the contrary, however, exosomes from infiltrated neutrophils into inflamed joints are protective for chondrocytes through a TGF-β1-mediated mechanism [102].

**Figure 2:**
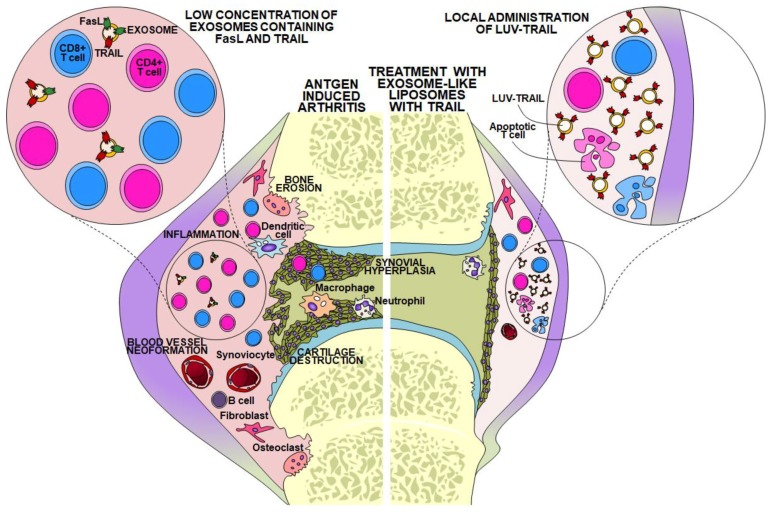
**Left**, schematic description of the situation in an arthritic lesion with the prototypical pathologic hallmarks: inflammatory infiltrate, blood vessel neo-formation, synovial hyperplasia, and, as a consequence, bone erosion and cartilage destruction. This situation is associated with a low concentration of regulatory FasL and/or TRAIL-containing exosomes, probably favoring T cell chronic infiltration. **Right**, situation upon intra-articular delivery of large unilamellar vesicles decorated with recombinant TRAIL (LUV-TRAIL), resulting in the elimination of synovial hyperplasia and in a reduction of the inflammatory infiltrate (based on data in references [103,105]).

Regulatory FasL- and TRAIL-containing exosomes produced by activated T cells present in the synovium could be beneficial to prevent autoimmune damage in rheumatoid disease. It Is known that T cells present in the synovium of RA patients have a chronically activated phenotype, but contrary to normal T cell blasts, are resistant to Fas-mediated apoptosis or growth inhibition signals [103,104,105]. However, our group showed that CD8^+^ T cells infiltrated in the synovium of RA patients were susceptible to recombinant TRAIL [104]. In addition, very low amounts of bioactive FasL or TRAIL associated with exosomes were found in the synovial fluids of RA patients, especially in the late stages of the disease [104]. This observation could account for the persistence of these T cells in spite of their sensitivity to TRAIL (see Figure 2, left). These data suggested that bioactive, membrane-bound TRAIL could be beneficial as an RA treatment. To verify this possibility we generated liposomes (large unilamellar vesicles, LUV) with a similar lipid composition as natural exosomes, to which recombinant TRAIL was fixed on their surface by using a Ni^+2^-bound coordination complex, termed LUV-TRAIL. These TRAIL-coated liposomes were then successfully used as a therapy in a rabbit model of arthritis, reducing macroscopic knee inflammation by 70%. The main effects of LUV-TRAIL in the inflamed tissue were the complete elimination of synovial hyperplasia, together with a substantial reduction of the inflammatory infiltration, both mononuclear and polymorphonuclear (Figure 2, right) [106]. The reduction of mononuclear infiltration could be related with the effect of the liposomes on T cells, but the impressive effect on synovial hyperplasia could be due to direct effects on synoviocytes or to indirect effects on cells that produce synoviocyte-stimulating cytokines, but this point was not addressed in that study. We must consider that TRAIL also induces the proliferation of certain populations of synoviocytes in RA [107,108], suggesting that it could be a double-edged sword in RA treatment. However, it should be still studied if proliferative effects are elicited only by soluble TRAIL and if membrane- or liposome-bound TRAIL, with a higher cross-linking efficiency, would rather induce apoptosis or cell cycle inhibition, as it has been demonstrated in tumor models [109,110,111]. 

In other studies in preclinical models, immunosuppressive exosomes produced by dendritic cells treated with IL-10, IL-4, or transfected with FasL also showed beneficial effects on rheumatoid disease [112,113,114]. In those studies, it was reported that the therapeutic mechanism, although mediated by death ligands, did not involve apoptosis. This could be probably due to cell cycle inhibition mediated by the induction of p21 expression in activated T cells by exosome-bound FasL and/or TRAIL [115,116,117]. This mechanism is also influencing the pathogenesis of autoimmune lymphoproliferative syndromes due to mutations in Fas or FasL and should be kept in mind for their treatment [115]. In fact, one of the most effective treatments in these syndromes, especially in severe cases, is rapamycin-based compounds affecting T cell cycle and proliferation [118,119].

### 3.2. Exosomes in Other Autoimmune and Chronic Inflammatory Diseases

Less information is available on the role of exosomes in the pathogenesis and their possible use as a treatment of other autoimmune or chronic inflammatory diseases.

In the case of multiple sclerosis (MS), it is known that exosomes can cross the blood-brain barrier and could thus contribute to spreading brain antigens to the periphery for their later presentation by antigen-presenting cells of the immune system [120]. However, it seems that exosomes generated physiologically in the Central Nervous System have a positive influence in tissue homeostasis, enhancing myelination and neuroprotection [120,121]. In any case, in an experimental autoimmune encephalomyelitis (EAE) murine model, it was clearly demonstrated that the injection of vesicles from microglial cells into the brain of mice developing the disease substantially increased its severity. In this line, mice deficient in acid sphingomyelinase, that show impaired EV secretion, were protected from EAE [122]. In addition, it has been shown that EVs derived from human brain microvascular endothelial cells are able to activate CD4^+^ and CD8^+^ T cells, probably contributing to autoantigen presentation [123]. These data, although limited, point to a pro-inflammatory role of EVs in MS when pathogenic conditions are favored.

Another pathology in which exosomes have been implicated is in chronic inflammatory lung disease [124]. In the case of chronic obstructive pulmonary disease (COPD), a very frequent pathology mainly caused by cigarette smoking, EVs derived from human lung tissue contain miR-210, which blocks Atg7 expression, preventing autophagy and causing myofibroblast differentiation and fibrosis [125]. In asthma, the expression levels of CD81, CD36 and HLA-DR in airway exosomes are increased [126]. The transfer of CD36^+^ exosomes would favor asthma progression by promoting inflammation through TLR4 and TLR6 complex formation [127]. Exosomes from bronco alveolar fluid of asthmatics contain functional leukotriene-producing enzymes, causing inflammatory mediator secretion by bronchial epithelial cells [128]. In this illness, exosomes produced by activated neutrophils or eosinophils infiltrated in the airway-surrounding tissue are also proinflammatory and supports the pathology [129].

In another allergic condition, contact hypersensitivity, it has been shown that tolerance induction to hapten-conjugated self-antigens was due to the secretion of exosome-like nanovesicles by T CD8^+^ suppressor cells, which contained miRNA-150 and inhibited the activation of effector T cells [130]. Later on, it was demonstrated the role of macrophages in this T CD8^+^ suppressor-mediated mechanism [131].

In type I diabetes, β pancreatic cells produce exosomes that contain autoantigens, favoring autoreactive T cell activation and disease [132,133,134]. Recently, a new mechanism of pathogenesis involving T-cell derived exosomes has been described [135]. In this study, the presence of specific miRNA and its transfer to pancreatic β cells led to β-cell death and expression of chemokine genes, that would increase in turn further infiltration of activated T cells.

The implication of exosomes in the pathogenesis of ulcerative colitis has been also suggested [136], but experimental data supporting this notion are scarce. The proteomic analysis of serum from dextran sulfate-induced acute ulcerative colitis in mice has given, however, some clues that point to this direction [137]. In this study, it has been shown that exosomes derived from these mice can induce activation of p38 and ERK in macrophages, leading to the active secretion of the pro-inflammatory cytokine TNF-α. In addition, the mentioned proteomic study demonstrated an increase in acute-phase proteins and in immunoglobulins able to activate complement in those exosomes with respect to those of normal mice. 

Finally, the presence of pro-inflammatory exosomes has been shown in the sera of systemic lupus erythematosus patients. These exosomes induce the secretion of TNF-α and IFN-α in PBMC through a TLR-mediated mechanism [138]. In addition, several studies suggest a pathogenic role for renal-derived exosomes in lupus nephritis [139]. 

The role of exosomes in the pathologies indicated above is summarized in Table 1.

It should be noted that most of the immunosuppressive mechanisms present in exosomes and that normally regulate tolerance induction are used by tumors to evade immune surveillance and are susceptible to therapeutic intervention. However, this extensively studied topic exceeds the purpose of this review.

## Figures and Tables

**Table 1:** Summary of the role of exosomes, depending on their procedence, on the development of the pathology indicated. +, exosomes favor pathology development; −, exosomes alleviate the pathology; ?, not known.

	Exosomes Produced by	Role in Pathology
Rheumatoid arthritis	Inflammatory infiltrate	+
	Synoviocytes	+
	Neutrophils	−
	T cells (death ligand containing; defective expression)	−
Multiple sclerosis	Physiological CNS tissue	−
	Activated microglia	+
	Brain microvascular endothelium	+
	T cells	?
Lung disease: COPD	Lung tissue from patients	+
Asthma	Airway tissue from patients	+
	Bronco alveolar fluid from patients	+
	Neutrophils/Eosinophils	+
	T cells	?
Contact hypersensitivity	T CD8^+^ suppressors	−
Type 1 diabetes	β pancreatic cells	+
	T cells	+
Ulcerative colitis	Inflamed intestinal tissue	+
	T cells	?
SLE	Sera	
Lupus nephritis	Renal tissue (urine)	+
